# All‐Inside Stabilization of Discoid Lateral Meniscus Popliteal Hiatus‐Related Instability Using Anterior Cord Release With Meniscus Retention

**DOI:** 10.1002/atn2.70360

**Published:** 2026-10-28

**Authors:** Seikai Toyooka, Haruki Miyazaki, Yutoshi Osaki, Noriaki Arai, Hirotaka Kawano, Takumi Nakagawa

**Affiliations:** ^1^ Department of Orthopaedic Surgery Teikyo University School of Medicine Tokyo Japan; ^2^ Department of Orthopaedic Surgery Ageo Central General Hospital Saitama Japan

## Abstract

Discoid lateral meniscus is a common cause of lateral knee pain, snapping, catching, and intermittent locking. Although saucerization combined with repair has been widely used, postoperative remodeling, reduced meniscal volume, extrusion, and degenerative changes on magnetic resonance imaging have raised concerns regarding meniscal resection. A meniscus‐retention concept using anterior cord release with robust suturing has been described, particularly for large displaced longitudinal tears with frank mechanical locking. In daily practice, however, many symptomatic cases present with popliteal hiatus‐related posterior peripheral disruption and popliteomeniscal fascicle insufficiency, resulting in peripheral rim instability and anterior translation of the discoid meniscus during knee motion. Here, we describe a simple and physiologic, meniscus‐retaining technique that combines anterior cord release to reduce anterior horn tension and facilitate peripheral reduction and targeted all‐inside vertical mattress fixation around the popliteus tendon. A pragmatic avoidance criterion is emphasized for extensive, notch‐locked displaced tears that likely require more robust fixation, in which inside‐out repair is preferred. This technique aims to control dynamic instability while preserving native meniscal tissue and minimizing intra‐articular environmental change.

## Graphical abstract

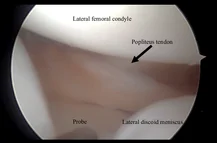

**VIDEO 1:** All‐inside stabilization with meniscus retention for popliteal hiatus‐related instability in a discoid lateral meniscus using anterior cord release. The video shows the following: (1) portal placement and diagnostic arthroscopy with an infrapatellar viewing portal in the figure‐of‐4 position; (2) dynamic probing to confirm peripheral rim instability around the popliteal hiatus and anterior translation of the discoid meniscus; (3) inspection of the popliteus tendon, popliteal hiatus, and the popliteomeniscal fascicle complex; (4) step 1—incremental anterior cord release extending to the level posterior to the anterior cruciate ligament tibial attachment to reduce anterior horn tension and facilitate peripheral reduction (no saucerization); (5) step 2—all‐inside vertical mattress fixation through the superior leaflet medial to the popliteus tendon using an all‐inside device (Truespan; DePuy Mitek, Raynham, MA); (6) step 3—creation of a far‐medial portal and placement of 2 additional superior‐leaflet vertical mattress sutures lateral to the popliteus tendon with continuous tendon visualization to avoid capture; and (7) final dynamic assessment confirming elimination of anterior translation and smooth gliding of the popliteus tendon within the sulcus, with discussion of conversion criteria to inside‐out repair for extensive displaced tears. Video content can be viewed at https://doi.org/10.1002/atn2.70360.

Discoid lateral meniscus (DLM) is a common cause of lateral knee pain, snapping, catching, and intermittent locking.^1^, ^2^ When peripheral instability is present, arthroscopic saucerization combined with meniscal repair has long been regarded as the standard surgical treatment.^3^, ^4^ Recently, however, increasing attention has been directed toward the potential drawbacks of meniscal resection in DLM. The discoid meniscus often undergoes postoperative remodeling, and follow‐up magnetic resonance imaging after trimming and repair may show a reduced meniscal volume and/or progressive degenerative changes.^5^, ^6^, ^7^ Altered load transmission after partial meniscectomy may contribute to chondral injury, progression of osteoarthritis, and even secondary osteochondritis dissecans.^8^, ^9^ Therefore, particularly in young and active patients, strategies aimed at preserving the native meniscal tissue and avoiding volume loss are desirable.

A meniscus‐retention concept was recently proposed by Suzuki and colleagues, in which anterior cord release is used to decrease excessive anterior hoop tension and facilitate peripheral reduction, followed by robust inside‐out suturing.^10^ This approach is particularly useful when a large, displaced longitudinal tear produces frank mechanical locking and requires strong fixation. In daily practice, however, we frequently encounter another symptomatic phenotype: a posterior tear around the popliteal hiatus with injury to the popliteomeniscal fascicle (PMF) complex, resulting in peripheral rim instability and anterior translation of the discoid meniscus during knee motion.^11^, ^12^, ^13^ In these cases, the primary pathology is dynamic instability rather than a massive meniscal tear, and overly rigid fixation may not be necessary.

Accordingly, we describe a meniscus‐retaining technique using an all‐inside device to stabilize symptomatic DLM with popliteal hiatus‐related posterior instability and anterior translation. By avoiding resection, this approach is intended to be physiologic and to minimize intra‐articular environmental changes while remaining simple and reproducible with widely available all‐inside devices.

## SURGICAL TECHNIQUE

### Surgical Indications

This meniscus‐retaining technique—combining anterior cord release with targeted all‐inside stabilization—is indicated for symptomatic DLM presenting with lateral pain, snapping, catching, or intermittent locking when arthroscopic evaluation reveals that the primary pathology is dynamic instability rather than a large displaced tear. Specifically, the technique is intended for cases with a posterior longitudinal tear around the popliteal hiatus and suspected dysfunction of the PMF complex, resulting in peripheral rim instability and anterior translation of the discoid meniscus. These findings should be confirmed by dynamic probing through an infrapatellar viewing portal with the knee in the figure‐of‐4 position. In terms of the Watanabe classification, this technique is most applicable to type III, or Wrisberg variant, DLM, in which posterior peripheral instability allows abnormal translation of the meniscus. We recommend avoiding this technique in cases that clearly require robust, extensive fixation—most notably when a large displaced longitudinal tear is completely locked into the intercondylar notch with frank mechanical locking. In such situations, strong fixation with a broader suture construct is typically necessary, and an inside‐out repair technique is generally more appropriate. In addition, this all‐inside, meniscus‐retaining approach should be used cautiously or avoided in menisci with extensive degenerative maceration, irreducible displacement despite anterior cord release, or when a safe device trajectory cannot be ensured because of inadequate visualization of the popliteus tendon and a heightened risk of tendon penetration or capture.

### Surgical Technique

The overall sequence of the procedure is shown in Video 1. The procedure is performed with the patient in the supine position under spinal or general anesthesia. A pneumatic tourniquet is applied to the proximal thigh and inflated at the discretion of the surgeon.

### Portal Placement and Diagnostic Arthroscopy

Standard anterolateral and anteromedial portals are established. A systematic diagnostic arthroscopy is performed to evaluate the patellofemoral joint, medial compartment, intercondylar notch, and lateral compartment. The lateral meniscus is palpated with a probe to assess morphology, tear configuration, and mobility.

With the knee in the figure‐of‐4 position, an en face view of the lateral compartment is obtained (typically through an infrapatellar viewing portal in this technique). The posterior horn of the lateral meniscus is translated anteriorly with a probe to confirm the presence of a DLM and characterize the tear pattern. Dynamic probing during knee motion is then performed to assess peripheral rim instability around the popliteal hiatus and confirm the anterior translation of the discoid meniscus that reproduces catching or intermittent locking (Figure 1).

**FIGURE 1:**
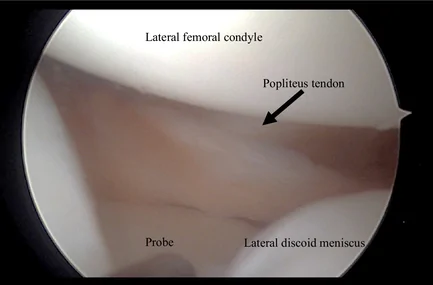
Arthroscopic image of the lateral compartment of the left knee viewed from the infrapatellar portal. When the discoid lateral meniscus is probed, the meniscus is easily translated anteriorly, indicating peripheral rim instability around the popliteal hiatus.

After confirming hiatus‐related instability, the popliteus tendon and the popliteal hiatus are inspected from the lateral compartment, with the knee brought toward extension as needed to optimize visualization. The PMF complex is assessed: the anteroinferior fascicle is identified as a fascial band extending from the inferior border of the lateral meniscus toward the posterior capsule along the popliteal hiatus, whereas the posterosuperior fascicle is visualized as a superiorly directed band arising from the posterior horn and inserting into the capsule over the popliteus tendon.^14^, ^15^ Findings suggestive of fascicle insufficiency include discontinuity or elongation of these bands and widening of the popliteal hiatus, which correlate with pathological meniscal mobility.

### Step 1: Anterior Cord Release (Tension Reduction)

While maintaining the arthroscope in the infrapatellar viewing portal, instrumentation is performed through the anterolateral portal. A scalpel is introduced, and a partial incision of the anterior horn is created. The cord‐like fibers posterior to the anterior cruciate ligament tibial attachment are carefully released until adequate tension reduction is achieved (Figure 2). Sufficient release is confirmed when the discoid meniscus opens laterally, the anterior horn tension decreases, and the peripheral rim shifts toward the capsule, facilitating reduction and decreasing the anterior traction force on the meniscus (Figure 3). No saucerization is performed. Insufficient release may leave persistent anterior translation and require excessive suture tension for stabilization, whereas over‐release may compromise anterior horn integrity and should be avoided by performing the release incrementally with repeated dynamic assessment.

**FIGURE 2:**
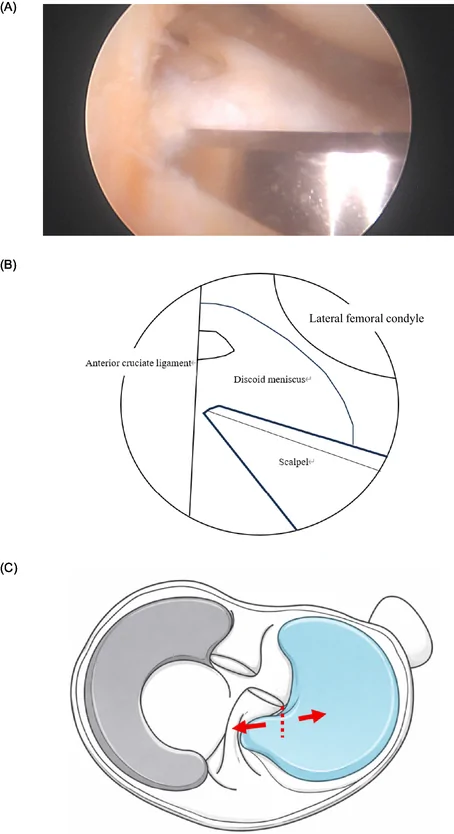
Arthroscopic image and illustrations of anterior cord release in the left knee. With the arthroscope in the infrapatellar portal, a scalpel is introduced and a partial incision of the anterior horn is created. The cord‐like fibers posterior to the ACL tibial attachment are carefully released until adequate tension reduction is achieved. Sufficient release is confirmed when the discoid meniscus opens laterally, anterior horn tension decreases, and the peripheral rim shifts toward the capsule, facilitating reduction and decreasing anterior traction on the meniscus: (A) arthroscopic view; (B) schema of anterior cord release; and (C) illustration of anterior cord release. (ACL, anterior cruciate ligament.)

**FIGURE 3:**
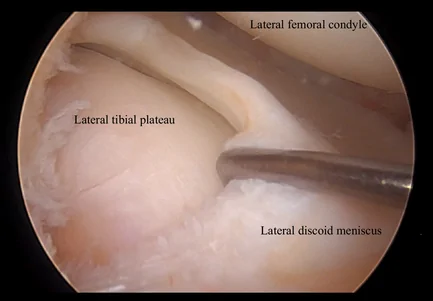
Arthroscopic image of the lateral compartment of the left knee viewed from the infrapatellar portal after anterior cord release. The discoid meniscus opens laterally, anterior horn tension decreases, and the peripheral rim shifts toward the capsule. These findings confirm sufficient release, improved reduction, and decreased anterior traction on the meniscus.

### Step 2: All‐Inside Fixation Medial to the Popliteus Tendon (1 Stitch)

Through the anteromedial portal, an all‐inside meniscal repair device (Truespan; DePuy Mitek) is introduced. A vertical mattress stitch is placed through the superior (femoral) leaflet of the meniscus medial to the popliteus tendon, aiming to restore functional restraint in the region corresponding to the posterosuperior PMF (Figure 4A). The suture is tensioned moderately (overtightening is avoided) and then cut. Because the discoid meniscus is often flat and thick, the device tip may have difficulty penetrating the meniscal surface. In such cases, using a more aggressively curved delivery option/guide may facilitate accurate device engagement. In addition, the suture travel path can be longer than that in a normal meniscus; therefore, the intended depth and distance to the capsule should be measured carefully with a probe (or depth gauge when available) before deployment to avoid overpenetration and ensure appropriate anchor placement.

**FIGURE 4:**
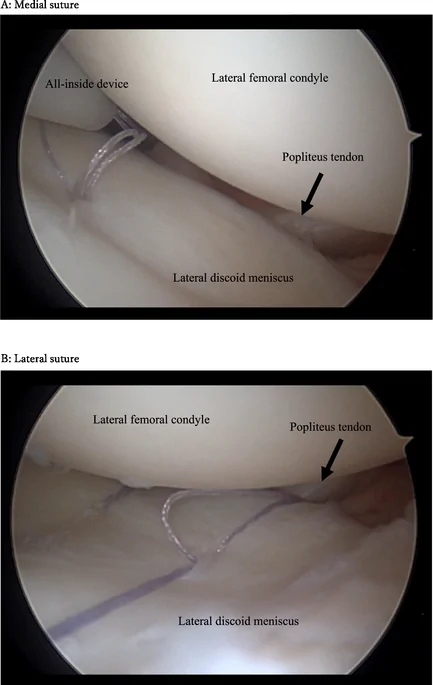
Arthroscopic images of the lateral compartment of the left knee viewed from the infrapatellar portal. The first all‐inside device is inserted through the anteromedial portal, and a vertical mattress suture is placed through the superior leaflet of the meniscus medial to the popliteus tendon. Subsequently, the second and third devices are inserted through the far‐medial portal, and 2 additional vertical mattress sutures are placed through the superior leaflet of the meniscus lateral to the popliteus tendon. The popliteus tendon should be carefully visualized to avoid tendon penetration or capture: (A) medial suture and (B) lateral suture.

### Step 3: All‐Inside Fixation Lateral to the Popliteus Tendon via a Far‐Medial Portal (2 Stitches)

A far‐medial portal is created to improve the approach angle to the lateral compartment. Using the same all‐inside device, 2 additional vertical mattress stitches are placed through the superior leaflet of the meniscus lateral to the popliteus tendon (Figure 4B). These sutures are placed to stabilize the unstable peripheral rim around the hiatus region and prevent recurrent anterior translation (Figure 5). Although the anteroinferior PMF lies inferior to the meniscus on the lateral side (and inferior‐leaflet fixation would be ideal), visualization of the inferior leaflet and the true peripheral rim is often limited in discoid menisci, making inferior‐leaflet passage a blind maneuver. Therefore, sutures are intentionally placed in the superior leaflet for safety and reproducibility.

**FIGURE 5:**
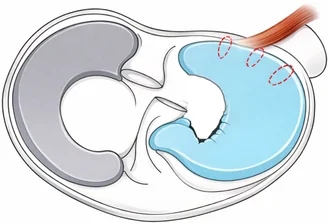
Illustration of the left knee showing all‐inside fixation around the popliteus tendon. Sutures are placed on the medial and lateral sides of the popliteus tendon to stabilize the peripheral rim while avoiding tendon capture or penetration. The exact suture locations are adjusted according to the tear pattern and area of maximal instability.

The popliteus tendon is continuously identified and directly visualized during all suture passages. Particular caution is required when placing sutures lateral to the popliteus tendon, where there is a higher risk of tendon penetration or capture. Because the popliteus tendon courses from superolateral to inferomedial, anchor deployment on the superolateral side of the tendon may place the implant in close proximity to the tendon. Therefore, the device trajectory and depth are carefully controlled under direct visualization to avoid iatrogenic tendon injury.

### Step 4: Final Assessment and Conversion Criteria

After fixation (commonly 1 stitch medial + 2 stitches lateral to the popliteus tendon), dynamic probing is repeated under infrapatellar portal portal visualization to confirm the elimination of anterior translation and resolution of catching/locking. The number and locations of sutures may be adjusted based on tear morphology; however, if ≥4 sutures are anticipated or if stability remains inadequate (especially in large displaced longitudinal tears with frank mechanical locking), conversion to inside‐out repair is recommended for more robust fixation. The knee is then taken through a full range of motion while visualizing the popliteal hiatus to confirm that the popliteus tendon sulcus has been effectively closed and that the tendon glides smoothly without impingement.

### Postoperative Rehabilitation

Postoperative rehabilitation is individualized according to patient factors and concomitant procedures; however, a typical protocol is as follows. Full weight‐bearing in extension with the use of crutches is permitted immediately after surgery. A knee extension brace is applied for the first 2 weeks to protect the repair and restrict range of motion. Controlled passive‐ and active‐assisted range‐of‐motion exercises are initiated after 2 weeks, with gradual progression as tolerated. Deep flexion beyond 90° in a weight‐bearing position is avoided for the first 3 months to minimize stress on the repaired PMFs.

Quadriceps and hamstring strengthening is started early with isometric exercises and then advanced to closed‐chain exercises as tolerated. Running is generally allowed at approximately 3 months postoperatively, whereas return to pivoting or cutting sports is typically permitted between 4 and 6 months, provided that muscle strength, neuromuscular control, and symptoms are acceptable.

## DISCUSSION

This technique offers a simple and physiologic solution for a clinical scenario that is frequently encountered in daily practice in DLM: anterior translation of the meniscus caused by posterior peripheral disruption around the popliteal hiatus, often associated with insufficiency of the PMF complex. Symptomatic DLM commonly shows peripheral rim instability, and the concept of “shift,” including anterior shift on magnetic resonance imaging, provides a useful framework to explain how meniscal mobility contributes to dynamic mechanical symptoms such as catching and intermittent locking.^16^

Compared with a normal meniscus, DLM has a larger morphology and broader coverage of the lateral tibial plateau, which may expose the entire lateral compartment to increased load and shear forces.^17^ This “size‐and‐shape” abnormality is consistent with the clinical observation that DLM is prone to meniscal tearing and peripheral detachment.^18^ Under substantial stress, the meniscocapsular junction may fail either anteriorly or posteriorly, resulting in anterior‐posterior instability and meniscal translation.^17^ In such a setting, simply repairing the detached rim to “restore the original position” may still leave the repaired site vulnerable to recurrent focal loading if the underlying tension environment remains unchanged.

Accordingly, our technique incorporates the meniscus‐retention anterior cord release concept described by Suzuki and colleagues as the initial step.^10^ By incrementally releasing the cord‐like fibers of the anterior horn, excessive anterior horn tension is reduced, the meniscus becomes easier to reduce, and the peripheral rim can shift toward the capsule. Suzuki et al. emphasized tension adjustment to reproduce meniscal reduction and physiologic motion while preserving the meniscus.^10^ We apply this philosophy to the phenotype of popliteal hiatus‐related posterior instability with anterior translation, aiming to control the dynamic “subluxation‐like” behavior of the discoid meniscus without meniscal resection.

Importantly, the benefit of this approach is not limited to “preservation” and avoidance of volume loss. Recent magnetic resonance imaging‐based studies after DLM treatment have reported postoperative morphologic remodeling with decreased residual meniscal width, as well as extrusion and degenerative signal changes, and extrusion has been associated with cartilage degeneration.^19^ These findings support the rationale for a meniscus‐preserving strategy that avoids excessive resection. Furthermore, we believe that anterior cord release may reduce the tensile and shear stress applied to the meniscocapsular repair site by improving reducibility and decreasing anterior traction. This stress‐reduction effect is a biomechanically plausible inference consistent with the concept of tension modulation described by Suzuki et al.; however, direct biomechanical validation remains an important topic for future investigation. Conceptually, creating a low‐tension environment first allows sutures to function primarily as stabilizers rather than as a means to forcibly “pull and hold” the meniscus in position, potentially decreasing the burden on the repair.

With respect to fixation strategy, it is essential to consider functional restoration of stability around the popliteal hiatus, where the PMF complex supports the posterior horn of the lateral meniscus adjacent to the popliteus tendon.^20^ In our method, a 3‐point construct is commonly used—1 vertical mattress stitch medial and 2 stitches lateral to the popliteus tendon, placed through the superior (femoral) leaflet. Although inferior‐leaflet fixation might theoretically better replicate the anteroinferior fascicle, visualization of the inferior leaflet and true peripheral rim is often limited in large discoid menisci, making inferior passage a blind maneuver and potentially unsafe. Therefore, we intentionally use superior‐leaflet sutures for safety and reproducibility. Because the risk of popliteus tendon capture is higher during lateral‐side suturing, continuous identification of the tendon and strict control of the device trajectory and depth are mandatory.

Finally, although this technique maximizes meniscal preservation with an all‐inside approach, appropriate case selection is critical. A 3‐point construct is sufficient in many cases, and the number and location of sutures can be adjusted to the tear morphology. However, for larger, displaced longitudinal tears with frank mechanical locking, or when broad, robust fixation is clearly required, an inside‐out repair remains a reliable option. Thus, our proposed algorithm offers a practical balance: treat the commonly encountered popliteal hiatus‐related anterior translation with a simple, physiologic, all‐inside stabilization with meniscus retention while reserving inside‐out repair for extensive tears that demand stronger fixation. The advantages and disadvantages of this technique are summarized in Table 1. Pearls and pitfalls are summarized in Table 2.

**TABLE 1:** Advantages and Disadvantages of the All‐Inside Stabilization With Meniscus‐Retention Technique for Popliteal Hiatus‐Related Instability in DLM

Advantages	Disadvantages
Meniscus retention (no saucerization): preserves native meniscal tissue and avoids volume loss	Not suitable for large displaced tears completely locked in the intercondylar notch; such cases typically require more robust fixation (prefer inside‐out)
Simple and physiologic concept: targets the common clinical phenotype of dynamic anterior translation because of popliteal hiatus‐related posterior instability	Inferior‐leaflet fixation is not routinely performed; anteroinferior PMF replication is limited because inferior passage is often not safely visualized in DLM
Tension modulation: anterior cord release improves reducibility and may reduce tensile/shear stress at the repair site (biomechanically plausible)	Risk of popliteus tendon penetration/capture, particularly when placing lateral sutures; requires continuous tendon visualization and strict depth control
All‐inside repair: efficient, avoids additional posterolateral incisions, and uses widely available devices	

DLM, discoid lateral meniscus; PMF, popliteomeniscal fascicle.

**TABLE 2:** Pearls and Pitfalls of the All‐Inside Stabilization With Meniscus‐Retention Technique for Popliteal Hiatus‐Related Instability in DLM

Pearls	Pitfalls
Perform anterior cord release incrementally and reassess after each small release to achieve adequate tension reduction. Insufficient release may require excessive suture tension and result in persistent translation; over‐release may compromise anterior horn integrity	Insufficient release may require excessive suture tension and result in persistent translation; over‐release may compromise anterior horn integrity
Measure the intended depth/distance to capsule with a probe (or depth gauge) before deploying all‐inside anchors because the suture travel path may be longer in DLM. Overpenetration can occur if depth is not measured, increasing the risk of iatrogenic injury and anchor malposition	Overpenetration can occur if depth is not measured, increasing the risk of iatrogenic injury and anchor malposition
If the discoid meniscus is flat and thick, consider a more aggressively curved guide/delivery option to facilitate device engagement	Forcing the device in a flat/thick meniscus may cause incomplete penetration, inaccurate placement, or iatrogenic tearing
Create a far‐medial portal to optimize the trajectory for lateral‐side sutures around the popliteal hiatus	Without a far‐medial portal, the approach angle may be poor, increasing the likelihood of popliteus tendon capture
	Popliteus tendon penetration/capture is a key hazard, especially laterally (superolateral anchor placement may be close to the tendon)

DLM, discoid lateral meniscus.

In summary, this meniscus‐retaining technique—combining anterior cord release with PMF‐oriented stabilization around the popliteal hiatus—provides a simple and physiologic option to control anterior translation of symptomatic DLM while preserving native meniscal tissue and minimizing intra‐articular environmental change.

## DISCLOSURES

The authors (S.T., H.M., Y.O., N.A., H.K., T.N.) declare that they have no known competing financial interests or personal relationships that could have appeared to influence the work reported in this article.
